# A combined measure of blood leukocytes, forced vital capacity and quantitative CT is highly predictive of mortality in IPF: results of a single-centre cohort study

**DOI:** 10.1186/s12890-025-03825-4

**Published:** 2025-07-28

**Authors:** Andrew Achaiah, Emily Fraser, Peter Saunders, Rachel Hoyles, Rachel Benamore, Ling-Pei Ho

**Affiliations:** 1 MRC Translational Immune Discovery Unit, Weatherall Institute of Molecular Medicine, University of Oxford, Oxford, UK; 2 Oxford Interstitial Lung Disease Service, Oxford University Hospitals NHS Foundation Trust, Oxford, UK; 3 Gloucestershire Hospitals NHS Foundation Trust, Cheltenham, United Kingdom; 4 Oxford Radiology Unit, Oxford University Hospitals NHS Foundation Trust, Oxford, UK

## Abstract

**Background:**

Idiopathic pulmonary fibrosis (IPF) is a progressive fibrotic condition. Serial FVC monitoring is most commonly used to assess progression of disease but FVC does not always reflect regional CT change in IPF. Recently there has been growing interest in quantitative CT (qCT) assessment of IPF. In this study, we compared different physiological and qCT measurements of disease progression in predicting mortality in IPF.

**Aims:**

We question if a composite measure of disease progression using qCT and FVC is more predictive of mortality than individual measurements, and if addition of blood leukocyte levels further enhance predictive ability of these measurements of disease progression.

**Methods:**

We conducted a retrospective analysis of an IPF cohort (*n* = 71). Annualised change (∆) in CT-measured lung volume (CTvol) and total lung fibrosis score (TLF) were calculated (using the computer software CALIPER) together with annualised change in FVC and blood leukocyte levels within 4 months of first CT. These were modelled against mortality using multivariate Cox regression. Concordance indexes (C-statistic) of different Cox regression models were used to determine the most predictive and discriminative combination for mortality.

**Results:**

65 cases (91.5%) were male. Median (IQR) age 73.6 years (68.4–79.3). Death was reported in 24 cases (33.8%). The median annualised change in (∆)FVC was − 4.4% (-9.6-0.0), ∆TLF; + 2.9% (0.2-7.0), and ∆CTvol; -4.3% (0.0-10.9). Combined measurements of disease progression (∆CTvol, ∆FVC and ∆TLF%) out-performed single-variable measurements in predicting all-cause mortality in IPF. The composite variable of [ΔFVC >10%, ΔCTvol >10% or ΔTLF% >10%] was most predictive of mortality [HR 7.14 (2.45–20.79), *p* <0.001]. Inclusion of blood leukocytes improved C-statistic scores for each multivariate model.

**Conclusion:**

Composite end points of ∆CTvol, ∆FVC and ∆TLF% were more predictive of mortality than single-variable measurements in this cohort. Inclusion of blood leukocytes into risk stratification models further improved mortality prediction for all measures of disease progression.

**Supplementary Information:**

The online version contains supplementary material available at 10.1186/s12890-025-03825-4.

## Key message

**What is already known on this topic:**

Idiopathic pulmonary fibrosis (IPF) is a behaviourally heterogeneous fibrotic condition. spirometry is most commonly used to assess progression of disease yet FVC is not always sensitive to regional morphologic changes observed radiologically in in IPF. Several human studies have implicated blood leukocytes (monocyte, neutrophil and lymphocyte) with disease progression and mortality.

**What this study adds:**

In this study, we compared different measurements of disease progression (annualised change in FVC, CT-measured lung volume and CT-measured total lung fibrosis score) in predicting mortality in IPF. The use of composite end points of disease progression captured more cases of progression and were more predictive of mortality than single-variable measurements in this cohort. Inclusion of blood leukocytes improved risk stratification, suggesting inclusion can improve the discriminative ability of measurements of disease progression for mortality.

**How this study might affect research, practice and/or policy:**

Assessing disease severity and accurately capturing disease progression in patients with IPF is a clinical priority that facilitates timely decision making regarding treatment initiation, assessment of treatment response and prognostication. In this context, the prospect of utilising easily measurable CT biomarkers could be an attractive adjunct to traditional FVC monitoring to capture cases of disease progression. Peripheral blood leukocytes have potential utility as a biomarker for identifying patients at greater risk of progression of IPF. This should be taken forward by prospective and validation studies.

**Supplementary Information:**

The online version contains supplementary material available at 10.1186/s12890-025-03825-4.

## Introduction

Idiopathic pulmonary fibrosis (IPF) is a progressive fibrotic condition with poor prognosis, with variable disease course [1]. In some individuals it advances with rapid lung function decline, whilst in others it progresses insidiously with periods of symptomatic and physiological stability [2].

Disease progression and prognosis are classically defined by decline in forced vital capacity (FVC) in international guidelines [3]. Annualised FVC decline >10% is considered highly prognostic of adverse outcomes [4], and in the UK FVC decline is used to guide antifibrotic prescribing [5]. However, FVC is subject to inter-test variability, which can leave marginal changes (5–10%) difficult to interpret [6]. Furthermore, patients might struggle to perform lung function due to fatigue or cough making comparison of results challenging [7]. Serial FVC measurement can be poorly sensitive to change, and does not always reflect regional morphological changes in geographically heterogeneous lung diseases such as IPF, as visualised by high resolution CT [8, 9]. Post hoc analyses of the PROFILE study suggest that FVC trajectories among IPF patients are heterogenous [10]. The need for tests more sensitive to disease progression in IPF is well recognised to facilitate accurate disease monitoring and timely treatment intervention [11].

Use of quantitative CT (qCT) assessment algorithms has been demonstrated to be highly prognostic in IPF and non-IPF fibrotic interstitial lung diseases [12–15], but can also enhance cohort enrichment for clinical trial end points [16]. However, prospective IPF studies demonstrate only moderate correlation between functional decline and radiographic assessment of progression of fibrosis [17]. Basic science studies have offered insight into the immune pathogenesis of IPF. Monocyte-derived macrophages, and neutrophil and lymphocyte sub-populations have been implicated in the immune-pathogenesis of lung fibrosis in both human and animal studies [18, 19]. Single-cell RNA sequencing has revealed changes within diverse cell populations during fibrosis [20]. These observations are supported by large-scale epidemiological studies that have identified peripheral blood monocytes, neutrophils and lymphocytes as potential biomarkers of progressive disease [21, 22].

In Achaiah et al., we previously reported (using quantitative CT assessment) that progression of fibrosis and reduction in CT-measured lung volume are associated with mortality in IPF. [23] In the same study we also demonstrated that neutrophil count measured in peripheral blood was associated with disease progression. We have also demonstrated that neutrophil count is associated with faster rate of FVC decline in IPF [24], and monocyte count to be associated with progression of interstitial lung abnormalities [25].

At a recent international symposium the need for study endpoints that most reliably capture whether emerging IPF therapeutics provide meaningful patient benefit was discussed [26]. Key to this is validating alternative measures of disease progression which could potentially shorten event-driven studies and provide accurate global reflection of disease burden [27]. Studies that have explored composite endpoints to prognosticate IPF are limited to changes in FVC and gas transfer, hospitalisation and mortality [27, 28]. To our knowledge composites that incorporate spirometry with quantitative CT assessment of fibrosis and lung volume to prognosticate IPF are relatively unexplored.

Therefore, using the same study cohort from Achaiah et al., [23] we explored (i) if composite endpoints of disease progression can improve prediction of mortality in IPF and (ii) whether inclusion of blood leukocytes into risk stratification models enhances mortality prediction. Some of the results of this study have been previously presented in abstract form [29].

## Methods

### Study design

We performed a retrospective analysis of a cohort of patients (National Health Service Research Ethics approval 14/SC/1060) with an MDT diagnosis of IPF [30]. 71 patients from the Oxford Interstitial Lung Disease (ILD) Service with baseline and follow-on high resolution thoracic CT (HRCT) scans compatible with the Computer-Aided Lung Informatics for Pathology Evaluation and Ratings quantitative CT tool (CALIPER) were included in the study (follow-up period September 2016 - November 2021).

Demographic profiles, lung function tests (FVC and transfer Factor of Lung for Carbon Monoxide (TLco)) closest to HRCT, and antifibrotic treatment duration were recorded. Absolute neutrophil, lymphocyte, and monocyte counts were taken from clinical ‘full blood count’ analysis (captured from electronic patient records) within 4 months of first CT scan [21, 23]. Lung function tests taken within 3 months of CT scan were recorded. Annualised change in FVC and CT metrics, and all-cause mortality rates were collected (full methodology is described in the online supplementary). Data acquisition has been previously described in Achaiah et al. [23]

### CT evaluation and CT visual assessment

Non-contrast, supine, volumetric HRCT scan were acquired using a 64-detector row CT scanner. Images were reconstructed using a high spatial resolution algorithm. Non-contrast, volumetric, high resolution CT scans for appropriate subjects were acquired (0.625 mm slice thickness at an interval of 0.625 mm) [23]. Visual assessment of CT has been previously described in Achaiah et al. [24] A thoracic radiologist with sub-specialty interest in ILD reviewed each patient’s high resolution thoracic CT at ILD MDT discussion to provide a radiological diagnosis that best correlated with each patient's clinical history. CT abnormalities were defined in accordance with standard Fleischner-based terminology and according to 2018 IPF guidelines [30, 31].

Follow-on CTs were classified as either ‘non-progressive’ or ‘progressive’ in comparison to initial CT. Cases were defined as ‘progressive’ if follow-on CT demonstrated either (i) visual (qualitative) increase in volume of existing disease or (ii) progression of UIP pattern. ‘Non-progressive’ cases were defined as unchanged pre-existing CT features at follow on CT scan either by way of (i) extent of disease or (ii) unchanged UIP pattern.

### CALIPER evaluation

#### Data processing

The CALIPER lung texture algorithm (v2.1) was acquired from IMBIO, Minneapolis, USA (www.imbio.com). As previously described in Achaiah et al., [23] initial data processing steps included (i) lung segmentation from adjacent thoracic and chest wall structures, (ii) separation of right and left lungs and (iii) airway segmentation. CALIPER-derived lung volume (CTvol) and total lung fibrosis scores (TLF) were obtained for each CT scan. Total lung volume (CTvol) was calculated as sum of right and left lung volume.

#### Pattern evaluation

CALIPER evaluation of CT data included classification and quantification of each volume of interest into one of six radiological parenchymal categories: normal lung, hyperlucent (areas of low attenuation), ground glass opacity (GGO), reticulation and honeycombing. Volumes for each parenchymal feature were expressed as a relative percentage of CALIPER-derived (i) total lung volume or (ii) zonal lung volume. Total lung fibrosis extent (TLF%) represented the sum of GGO, reticular and honeycomb percentages [23].

#### Pulmonary function tests

Spirometry (FEV1 and FVC), and Transfer Factor of the Lung for Carbon Monoxide (TLco) were captured closest to CT. Values are expressed as absolute (FVC; litres, TLco; mmol/min/kPa) or as percentage of predicted value. To draw meaningful comparison to CT trends, annualised trend in lung function trends were derived from lung function tests recorded closest to first and second CT scan.

### Measurement of disease progression

Changes from baseline absolute FVC, TLF and CTvol between baseline and follow-on CT scans were calculated as an annualised metric (∆). Disease progression was defined as either a continuous or categorical annualised metric, or as a composite of ∆FVC, ∆TLF and ∆CTvol. Annualised percentage change from baseline to follow-up investigation was calculated as:

$$\small \tiny \large \small \tiny \scriptsize \small \scriptsize \:Annualised\:percentage\:change\:(\varDelta\:)=\frac{\left[\right(Absolute\:difference\:\div\:Variable\:at\:baseline)\:\times\:\:100]}{Time\:interval\:between\:baseline\:and\:follow\:up\:investigation\:\left(Years\right)}$$

Annualised change was expressed as continuous or categorical variables (>5% or >10%). Composite metrics were made up of different combinations of categorised ∆FVC, ∆TLF and ∆CTvol.

### Statistical analysis

Pearson correlation was used to explore association between baseline and annualised CALIPER-variables and pulmonary function tests. ∆FVC, ∆TLF and ∆CTvol and composite variables were compared against mortality using Cox regression. The censoring time for mortality assessment was the end of follow-up period; 1st January 2022. Multivariate models were adjusted for age, gender, baseline Total lung fibrosis score (TLF%), antifibrotic duration and leukocytes (absolute monocyte, neutrophil and lymphocyte count). Multivariate models were tested with inclusion and exclusion of leukocytes and Harrell’s Concordance index (C-statistic) was used to determine the most discriminate models for predicting mortality (assess model strength, describing how well a model can discriminate between two survival distributions) [32].

Data are expressed as absolute values, relative percentages, means (with standard deviation), medians (interquartile range) or by dichotomised value. Normality testing was performed using a D’Agostino & Pearson test and following this difference between groups was analysed using either paired or unpaired t-tests or Mann-Whitney test for respective parametric and non-parametric analysis. Fisher’s exact test of significance was used to assess differences in categorical data.

Statistical confidence intervals (CI) are at 95%, where reported. Two-tailed p values <0.05 determined statistical significance. All analyses were performed using Graphpad Prism (version 9.2) or SPSS version 27 (IBM Armonk, NY, USA). UpSet plots were used to visualise the relationship and proportions of categorical measurements of disease progression in this cohort using UpSetR Shiny App (https://gehlenborglab.shinyapps.io/upsetr/).

#### Patient and Public Involvement

Patient and public were not involved in design, recruitment, conduct of this study.

## Results

### Demographic

71 cases with CALIPER compatible baseline and follow on CT scans were analysed. Demographic profiles are displayed in Table 1. Median (IQR) time interval between CT scans was 25.9 (16.8–39.9) months. Time between CT and closest lung function test was 0.23 (−1.45 to 2.33) months. Time between CT1 and blood draw; −0.95 (−2.47 to 2.26). Median length of follow-up from CT1 for all cases was 41.6 (18.1–52.0) months. Median time from first CT to death was 31.6 months (24.7–45.7) and 48.5 (39.9–61.5) in those surviving to censoring date. Antifibrotic use was greater in patients that died during follow up (*p* = 0.326), and median duration of antifibrotic use was shorter in comparison to those that survived (*p* = 0.327). Although neither finding was statistically significant.

**Table 1:** Baseline characteristics

Patient characteristics	All patients (*n* = 71)	Survived (*n* = 47)	Death (*n* = 24)
**Demographics**			
** Age at CT**	73.5 (68.3–79.4)	73.6 (68.4–79.2)	72.9 (67.2–81.0)
** Male**	65 (92%)	41 (88.2%)	24 (100%)
** Ex-smoker**	38 (50.6%)	26 (55%)	11 (45.8%)
**Co-morbidities**			
** Reflux**	25 (39.7%)	15 (35.1%)	10 (52.6%)
** PHT**	4 (6.3%)	3 (6.8%)	1 (5.3%)
** IHD**	15 (23.8%)	11 (25.0%)	4 (21.1%)
** Cardiomyopathy**	1 (2.0%)	1 (2.6%)	0 (0.0%)
** Arrhythmia**	2 (3.2%)	0 (0.0%)	2 (10.5%)
** Hypertension**	16 (25.4%)	13 (29.5%)	3 (15.8%)
** PE**	2 (3.2%)	1 (2.3%)	1 (5.3%)
** COPD/Emphysema**	4 (6.3%)	2 (5.3%)	2 (18.2%)
** Type II diabetes mellitus**	7 (11.1%)	6 (13.6%)	1 (5.3%)
**HRCT pattern**			
** Probable UIP**	31 (41.9%)	24 (48%)	7 (17%)
** Definite UIP**	43 (58.1%)	26 (52%)	17 (70.8%)
**CALIPER CT assessment**			
** CT Lung volume (L)**	4.00 (3.70–5.10)	4.00 (3.70–5.10)	3.85 (3.65–4.80)
** Low attenuation area (%)**	1 (0–3)	1 (0–2)	1 (0–5)
** Total Ground Glass Opacification (%)**	5 (2–10)	4 (2–9)	10 (3–14)
** Total Reticulation (%)**	5 (3–8)	5 (3–7)	5 (4–8)
** Total Honeycombing (%)**	0 (0–1)	0 (0–1)	0 (0–1)
** Total lung fibrosis (%)**	13 (7–19)	11 (7–16)	15 (9–23)
** Upper Zone fibrosis (%)**	5 (1.5–11)	4.5 (1.0-8.5)	10.3 (2.8–20.3)
** Middle Zone fibrosis (%)**	8.5 (2.5–18.5)	8.5 (4.0–16.0)	9.3 (1.3–22.3)
** Lower Zone fibrosis (%)**	24 (11.5–42.5)	22 (12.0–42.0)	32.0 (6.3–46.3)
**Pulmonary function**			
** FEV1 (L)**	2.34 (1.93–2.83)	2.34 (2.06–3.05)	2.30 (1.80–2.64)
** FEV1 (%)**	84.7 (71.5–91.2)	86.5 (76.0-95.5)	75.35 (66.4–90.0)
** FVC (L)**	2.91 (2.35–3.56)	2.95 (2.41–3.53)	2.86 (2.26–3.70)
** FVC (%)**	76.4 (68.9–92.4)	76.9 (69.6–94.1)	73.4 (65.6–91.7)
** FEV1:FVC ratio**	0.82 (0.75–0.88)	0.83 (0.75–0.88)	0.80 (0.74–0.87)
** TLCO (mmol/min/kPa)**	4.90 (3.87–5.80)	5.56 (3.95–6.17)	4.46 (3.64–5.18)
** TLCO (%)**	60.1 (51.1–71.0)	64.0 (57.0-76.2)	52.3 (46.9–57.4)
** Time between CT and PFTs (months)**	1.08 (−1.31 to 2.92)	1.08 (−1.32 to 3.87)	1.04 (−1.02 to 2.83)
**Blood leukocytes**			
** Monocyte (x10**^3^**/µl)**	0.66 (0.55–0.80)	0.64 (0.55–0.77)	0.73 (0.55–0.80)
** Neutrophil (x10**^3^**/µl)**	4.87 (3.85–6.45)	4.72 (3.77–5.87)	5.34 (4.26–6.75)
** Lymphocyte (x10**^3^**/µl)**	1.64 (1.43–2.27)	1.73 (1.45–2.43)	1.51 (1.25–2.14)
** Time between CT and blood draw (months)**	0.95 (−2.47 to 2.26)	0.93 (−2.53 to 2.26)	1.01 (−2.46 to 2.30)
**Antifibrotics**			
** Antifibrotic use (n**,**%)**	36 (49.3%)	22 (44.9%)	14 (58.3%)
** Antifibrotic duration (months)**	63.5 (22.3-101.5)	68.8 (35.5-119.2)	44.5 (17.0-91.3)

All values are median (IQR) unless stated. Low attenuation areas represent emphysematous areas. CALIPER CT evaluation displayed as a as % of CALIPER measured CT-lung volume.

**Fig. 1:**
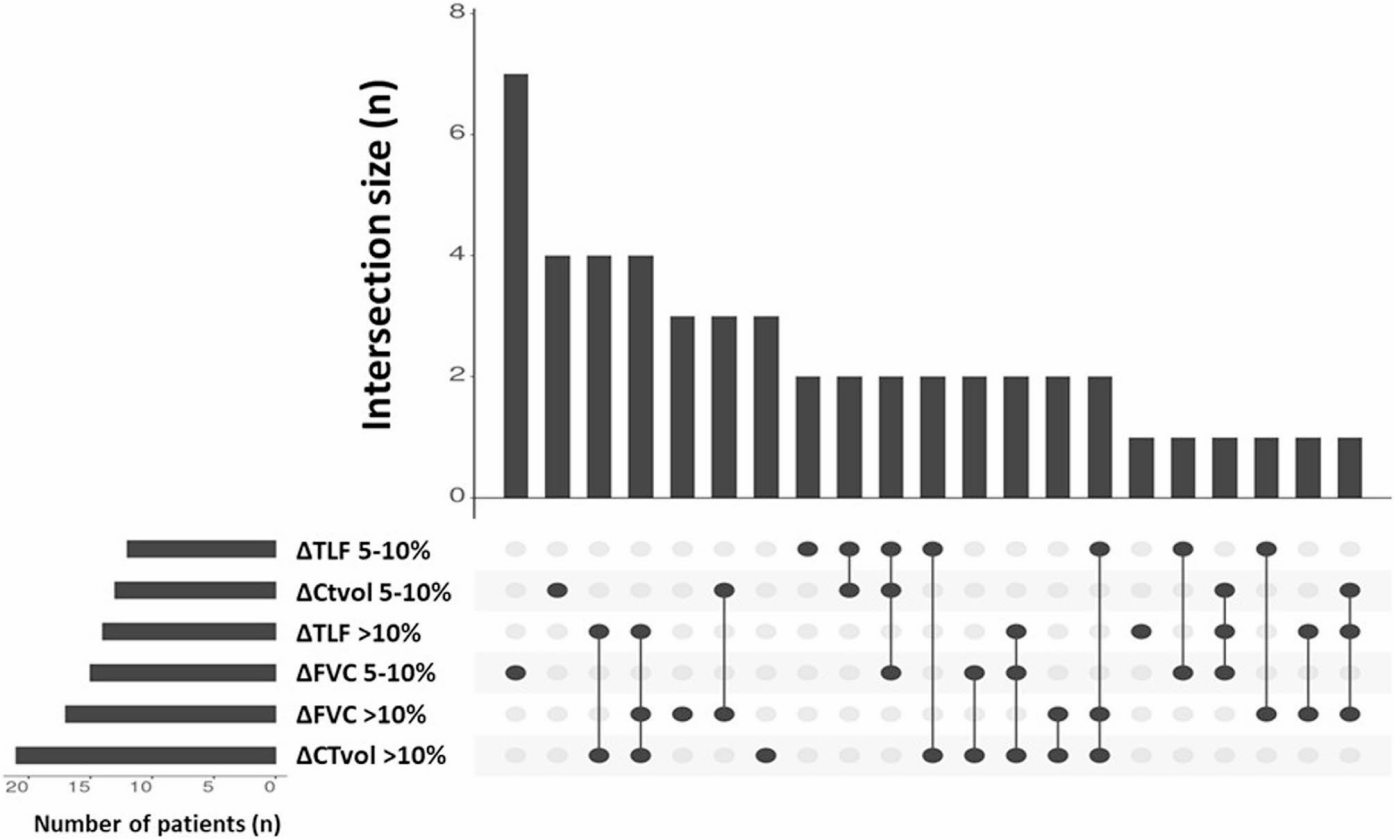
UpSet plot illustrating co-occurrence of different measurements of disease progression (∆CTvol, ∆TLF, ∆FVC). Number of patients corresponds to number of participants with each specific metric of disease progression. Intersection size refers to the number of patients with only those features indicated

Median values for annualised (∆) progression were as follows: ∆FVC − 4.4% (−9.6 to 0.0), ∆TLF 2.9% (0.2-7.0), ∆CTvol − 4.3% (0.0-10.9). The co-occurrence of different measurements of disease progression are illustrated by the UpSet plot in Fig. 1.

### CT and lung function measurements at baseline and at follow-on CT

We used Pearson r correlation to assess relationship between quantitative CT and FVC. There was significant correlation between baseline CT and FVC measurements; absolute FVC and CTvol (*r* = 0.668, *p* <0.001), FVC% and TLF% (*r *= −0.383, *p* <0.001), and CTvol with TLF% (*r *= −0.443, *p* <0.001). Of the annualised measurements of disease progression, there was significant, but weak, correlation between ∆TLF and ∆CTvol ( *r*= −0.277, *p* = 0.017), but not between ∆FVC with ∆TLF (*r *= −0.123, *p* = 0.316) or ∆FVC with ∆CTvol (*r* = 0.230, *p* = 0.057).

### Association between combinations of disease progression measurements and mortality

**Fig. 2:**
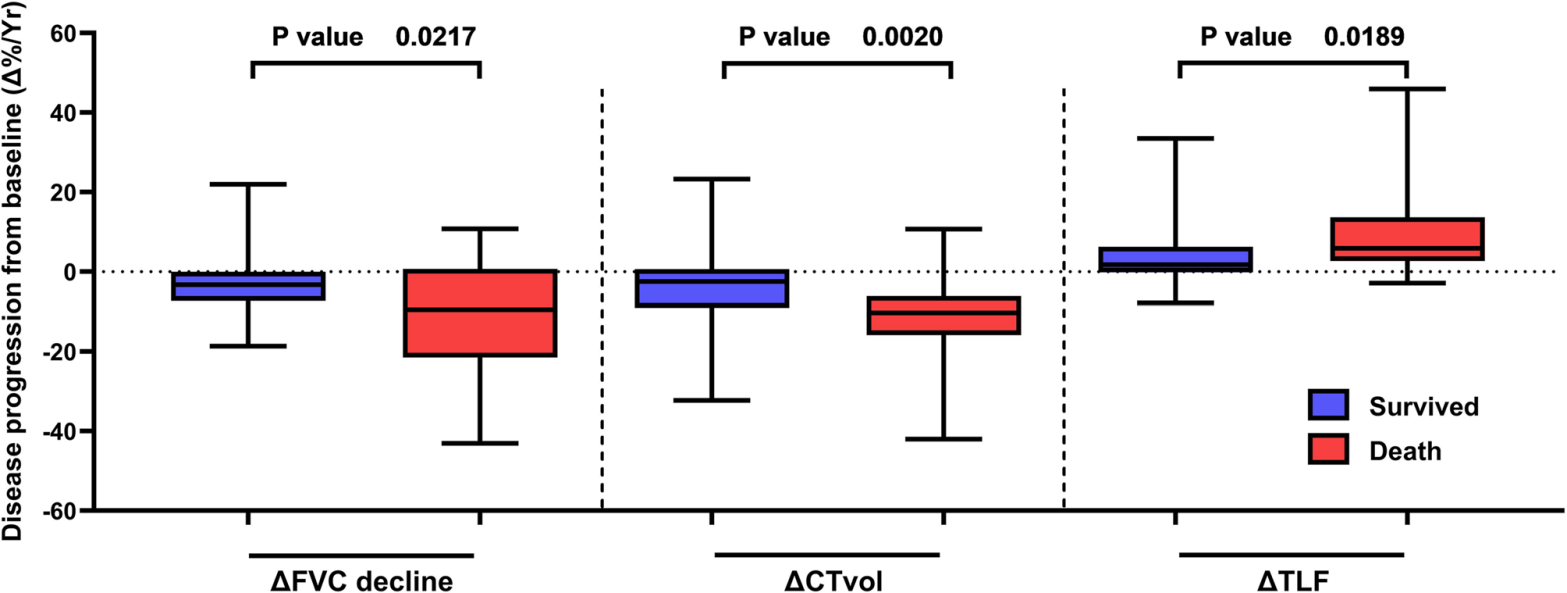
Box plots illustrating progression of disease measured against survival. Disease progression defined as reduction in FVC (∆FVC), CT volume (∆CTvol) and increase in Total Lung Fibrosis score (∆TLF)

We explored association between single-variable FVC, CTvol and TLF and composite measurements of disease progression with mortality. Disease progression was significantly worse in the group of patients that died during follow up; ∆FVC −10.5% vs. −3.4%, *p* = 0.022, ∆TLF 5.32% vs. 1.69%, *p* = 0.019, ∆CTvol −9.96% vs. −2.99%, *p* = 0.002. See Fig. 2 and Figure S2.

Table 2 describes the proportion of cases with disease progression measured by a categorical change of >5%/yr or >10%/yr. Multivariate Cox regression analysis is also described in Table 2. In Total, 24 patients died during follow-up (33.8%). ∆FVC >10% was the single variable measurement most predictive of mortality [HR 6.15 (95%CI 2.31–16.39), *p* < 0.001]. Yet of the 24 patients that died, ∆FVC >10% was reported in only 11 (46%). Of the remaining 14 cases, 10 demonstrated disease progression as either decline in CTvol >5% or increase in TLF > 5%.

**Table 2:** Multivariate Cox regression for the outcome of mortality

					Models not including leukocytes			Models including leukocytes		
Model	**Measurement of progression**	**Patients with measurement of progression n (%)**		**Mortality during follow-up n (%)**	**HR (95% CI)**	***P *Value**	**C-statistic**	**HR (95% CI)**	***P*Value**	**C-statistic**
1	Visual assessment of progression		40 (65.6)	15 (21.1)	1.69 (0.52–5.45)	0.381	0.724	1.98 (0.49–8.06)	0.338	0.806
2	ΔCTvol %		--	--	1.05 (1.01–1.08)	0.009*	0.740	1.05 (1.01–1.10)	0.012*	0.766
3	ΔCTvol >5%		36 (48.6)	18 (25.4)	2.78 (0.99–7.78)	0.052	0.730	3.53 (1.21–10.27)	0.021*	0.790
4	ΔCTvol >10%		23 (31.5)	12 (17.0)	2.95 (1.26–6.93)	0.013*	0.747	3.86 (1.51–9.87)	0.005*	0.792
5	ΔTLF %		--	--	1.03 (0.99–1.09)	0.170	0.717	1.04 (1.00-1.09)	0.058	0.785
6	ΔTLF >5%		28 (38.4)	13 (18.3)	2.30 (1.01–5.27)	0.049*	0.723	2.43 (1.00-5.89)	0.049*	0.786
7	ΔTLF >10%		14 (20%)	7 (9.9)	2.37 (0.94–5.96)	0.067	0.760	2.02 (0.79–5.15)	0.143	0.770
8	ΔFVC %		--	--	1.01 (1.00-1.03)	0.083	0.786	1.01 (1.00-1.03)	0.106	0.797
9	ΔFVC >5%		33 (47.8)	15 (21.1)	3.70 (1.44–9.48)	0.006*	0.758	4.79 (1.70-13.48)	0.003*	0.818
10	ΔFVC >10%		17 (24.6)	11 (15.5)	6.15 (2.31–16.39)	<0.001*	0.811	10.7 (3.45–33.23)	< 0.001*	0.866
11	ΔFVC >5% or ΔCTvol >5% or ΔTLF% >5%		52 (73.0)	21 (29.6)	4.24 (1.02–18.39)	0.048*	0.728	7.09 (1.50-33.56)	0.014*	0.833
12	ΔFVC >10% or ΔCTvol >10% or ΔTLF% >10%		33 (46.0)	18 (25.4)	7.14 (2.45–20.79)	< 0.001*	0.805	8.22 (2.71–24.94)	< 0.001*	0.851
13	ΔFVC >5% & ΔCTvol >5% & ΔTLF >5%		12 (16.4)	8 (11.3)	2.46 (1.01–5.98)	0.047*	0.747	2.58 (1.03–6.46)	0.043*	0.784
14	ΔFVC >10% & ΔCTvol >10% & ΔTLF >10%		4 (5.5)	3 (4.2)	6.70 (1.81–24.81)	0.004*	0.757	5.22 (1.23–22.12)	0.025*	0.793
15	ΔFVC >5% & ΔTLF >5%		15 (20.5)	9 (12.7)	2.70 (1.13–6.47)	0.025*	0.758	3.02 (1.21–7.54)	0.018*	0.792
16	ΔFVC >10% & ΔTLF >10%		6 (8.2)	4 (5.6)	7.09 (2.12–23.68)	0.001*	0.753	5.63 (1.61–19.69)	0.007*	0.801

Multivariate Cox regression for *n* = 71 patients. Progression defined by annualised ΔFVC, ΔTLF and ΔCTVol categories. Hazard ratios in multivariate model generated with or without inclusion of leukocytes (monocyte, neutrophil and lymphocyte counts), and adjusted for age, gender, low attenuation area, antifibrotic duration and baseline CALIPER TLF score. *; *p*<0.05 considered statistically significant

The composite variable of [ΔFVC >10%, ΔCTvol >10% or ΔTLF% >10% (Model 12)] was most predictive of mortality [HR 7.14 (2.45–20.79), *p* < 0.001]. The composite of [ΔFVC >5%, ΔCTvol >5% or ΔTLF% >5% (Model 11)] captured the most cases of disease progression (*n* = 52, 73%) and mortality (21, 29.6%), and was significantly associated with mortality in this cohort [HR 4.24 (1.02–18.39), *p* = 0.048].

Of this cohort, 40 patients (65.6%) demonstrated progression as measured as visual assessment of progression of fibrosis using CT scan. Mortality was observed in 15 cases (21.1%). Despite this, visual assessment of disease progression was not predictive of mortality [HR 1.69 (0.52–5.45), *p* = 0.381].

C-statistic scores were used to compare the predictive ability of each multivariate model for mortality based upon different composite measurements of disease progression. The inclusion of blood leukocytes (monocytes, neutrophils and lymphocytes) into multivariate models across all categories of disease progression (single variable and composite) appeared to enhance C-statistic scores for each model. The highest C-statistic score was observed when ΔFVC >10% was used to define disease progression in multivariate modelling.

## Discussion

In this study, the composite metrics of decline in forced vital capacity (ΔFVC), change in CALIPER-measured lung volume (ΔCTvol), and change in CALIPER-measured total lung fibrosis score (ΔTLF) were more predictive of mortality than respective single-variable categorical measurements. Addition of monocyte, neutrophil and lymphocyte count to risk stratification models further enhanced disease progression hazard ratios for mortality and the overall ability of multivariate models to predict mortality.

Composite endpoints aggregate multiple outcomes into a single endpoint. Potential advantages to clinical studies include enhanced statistical efficiency due to higher event rates, smaller sample size to demonstrate effect and shorter duration of event-driven studies. In clinical practice, adoption of composite endpoints could enhance prognostication for adverse clinical outcomes and assessment of therapeutic responses [27].

In chronic and progressive diseases such as IPF, which manifest with a multitude of sequelae, composite endpoints enable different clinically important domains to be captured. The utility of composite endpoints was discussed recently at an international symposium, and at which recent negative phase III IPF studies were critiqued [26, 33]. Discussants highlighted the possibility of substituting FVC as the primary outcome measure with a composite endpoint. However, deciding upon the appropriate components with which to build a composite requires further assessment and validation. There should be minimal co-linearity between components and appropriate weighting should be applied to each component [34]. Use of composite endpoints to study IPF is limited to studies defining composites by pulmonary function tests, hospitalisation and mortality [22, 35–40].

To our knowledge, our study is the first to assess a composite defined by longitudinal FVC and quantitative CT measurements. By defining disease progression as a composite a higher number of (i) disease progression events and (ii) mortality events were observed during follow-up. Therefore patients meeting any specified form of disease progression in our study were considered higher risk of mortality. Although ∆FVC >10% was most predictive of mortality and exhibited greatest C-statistic score, this metric captured fewer progression (17) and mortality events (11) in comparison to composites. [ΔFVC >5% or ΔCTvol >5% or ΔTLF% >5%] captured most cases of progression (52) and mortality (21). ΔCTvol, although a weaker predictor of mortality in comparison to ΔFVC, did capture more cases of disease progression (including 9 cases without progression-defining FVC decline), which is comparable to other studies [41].

We observed weak correlation between FVC decline and quantitative CT (qCT) measurement of lung volume decline (∆CTvol) and progression of fibrosis (∆TLF). Other studies have observed similar findings. In a retrospective analysis of placebo cohorts from IPF and Systemic sclerosis studies Zou et al. demonstrated weak association between baseline qCT metrics and FVC slope. [42] These observations are important as the apparent lack of co-linearity between longitudinal change in FVC with fibrosis score and lung volume may support inclusion of qCT parameters within composite metrics.

The reason for the greater predictive ability of composite endpoints observed in this study is not immediately clear. It could be because progression of fibrosis and loss of lung volume in IPF (∆TLF and ∆CTvol) do not correspond to immediate, simultaneous, decline in FVC. Studies exploring the relationship between FVC and gas transfer in IPF may provide insight. In a cross-sectional study, Cortes-Telles et al. (2014) demonstrated 50% of patients have preserved FVC at IPF diagnosis, yet display a restrictive pattern (measured by total lung capacity). [43] In a longitudinal IPF study by Soumagne et al. (2023), 1/3rd of patients demonstrating significant longitudinal decline in TLco did not demonstrate significant FVC decline. [9] Therefore, perhaps it is later in disease course and when fibrosis extent is sufficient to impair lung elastic recoil, that FVC decline is likely to be observed [9].

Spirometry is currently the most commonly used tool for assessing disease progression and treatment response in IPF, however it is not without limitation. Although FVC decline >10%/year is a validated predictor of mortality [4], longitudinal data demonstrate substantial intra-patient variability [6]. In post hoc analysis of the INSPIRE study, Cottin et al. concluded that in IPF patients with considerable emphysema, a stable longitudinal FVC was not representative of stable disease. [44] Furthermore, COPDGene post hoc analysis demonstrated progression of fibrotic interstitial lung abnormalities in patients with a stable FVC carried greater mortality risk [45]. This might imply that small increases in fibrosis represents nascent disease might not be captured by FVC.

Assessment of both disease severity and progression in IPF is a clinical priority to facilitate timely treatment initiation, assessment of response, and prognostication. From a research perspective, studies require novel sensitive markers to measure response to study interventions. This is even more pressing given the role of antifibrotic agents in delaying FVC decline [26]. Prospective studies could explore if clinical trial endpoints combining these can improve our definition of disease progression and treatment response in ILD.

Blood leukocytes have been implicated in the pathobiology of IPF [46]. We have previously demonstrated association between blood leukocytes and mortality in IPF [47], faster rate of FVC decline [24], and progression of fibrosis [23]. In those publications we discussed the potential mechanistic relationship between blood leukocytes and adverse clinical outcomes in IPF observed in basic science and epidemiological studies [21, 22, 48–51].

In this particular study we explored how absolute blood leukocytes can influence the ability of risk models in IPF to predict outcome by comparing C-statistic scores. The C-statistic score is measure of how well a model can predict the risk of an outcome [32]. Inclusion of leukocyte values into predictive models improved C-statistic scores and enhanced risk stratification. Kreuter et al. previously demonstrated in post hoc analysis of the ASCEND and CAPACITY phase III placebo-controlled trials that inclusion of peripheral blood monocyte count into a modified gender-age-physiology (GAP) model improved outcome prediction. [52] Both studies demonstrate the potential utility of leukocytes to model prediction in IPF independent of other model components.

Our findings should be framed with the limitations of a retrospective single-centre study. The retrospective nature of the study meant that we selected patients who had repeat CT scans, which were clinically indicated. This would have limited sample size and also introduced a selection bias towards patients who had a clinical reason to have a CT scan– often, this is worsening of disease, and CT scans would be performed at different/non-uniform time intervals. Each measure of disease progression in composites was considered to have equal importance which may not be true. Further evaluation of larger prospective studies will be required to validate our findings, and to ascertain appropriate weightings of each component of any future composites [53].

From the point of fibrosis scoring, the sum of GGO, reticulation and honeycombing was used as the total lung fibrosis score [54]. Although each scan was reported by a radiologist who ‘called’ the GGO as fine fibrosis and the overall pattern as probable or definite UIP, other causes such as pulmonary oedema, acute exacerbation and infection could have contributed to this CT finding [55]. We also acknowledge anti-fibrotic treatment may have affected blood leukocyte measurement and disease trajectory. 36 (49.3%) patients that underwent a second CT scan were receiving antifibrotics at first CT scan, and this may have biased the observed association. However multivariate analysis was adjusted for anti-fibrotic duration.

Visual assessment of progression of fibrosis was not predictive of mortality in this cohort. Over 65% of this cohort demonstrated progression of fibrosis upon radiologist assessment. This may highlight inter-reporter variability in subjective comparison of serial CTs. Such a limitation has been previously reported in the literature [56, 57], however all CTs were reviewed by experienced ILD radiologist during MDT discussion. Any bias towards indication for second CT scan may have also influenced this.

Change in FVC over time was calculated as the annualised change between two pre-defined time points (CT1 and CT2). Due to limitation of available FVC data in this cohort we were unable to measure the ‘slope of change’ from multiple values, which may have limited any intrinsic variability of the test. However time interval between lung function tests and CT scan were relatively short (median 0.23 months). Furthermore, in multivariate analysis all metrics were compared against all-cause mortality, which has long been considered a robust clinical endpoint in IPF [28, 58].

We acknowledge that the composite endpoints explored in this study do not provide further additional information on regional morphological changes in fibrosis, however it does provide a more comprehensive global picture of disease burden in this patient cohort. Further evaluation of larger prospective studies will be required to validate our findings and explore the optimal components of a disease progression composite metric to prognosticate in IPF.

In conclusion, our study shows that composite measurement of disease progression using FVC and quantitative CT metrics out-performs single-variable measurements in predicting all-cause mortality in IPF in this cohort. Inclusion of blood leukocytes into risk models enhanced model prognostication. The utility of both of these should be explored in future studies.

## Supplementary Information

Supplementary Material 1.

## Data Availability

All data generated or analysed during this study are included in this published article and supplementary information files.
