# Supplementary material for: A combined measure of blood leukocytes, forced vital capacity and quantitative CT is highly predictive of mortality in IPF: results of a single-centre cohort study
Source: BMC Pulm Med. 2025 Jul 28;25:358. doi: 10.1186/s12890-025-03825-4 (PMC12302753; doi:10.1186/s12890-025-03825-4)
Supplement: Supplementary file 1 — Supplementary Material 1. [file 12890_2025_3825_MOESM1_ESM.docx]

# **A combined measure of blood leukocytes, forced vital capacity and quantitative CT is highly predictive of mortality in IPF: results of a single-centre cohort study**

**Andrew Achaiah^1,2^, Emily Fraser^2^, Peter Saunders^2^, Rachel Hoyles^2^, Rachel Benamore^3^, Ling-Pei Ho^1,2^**

1. MRC Translational Immune Discovery Unit, Weatherall Institute of Molecular Medicine, University of Oxford, Oxford, United Kingdom.

2. Oxford Interstitial Lung Disease Service, Oxford University Hospitals NHS Foundation Trust, Oxford, United Kingdom.

3. Oxford Radiology Unit, Oxford University Hospitals NHS Foundation Trust, Oxford, United Kingdom.

**Correspondence to:**

Dr Andrew Achaiah

MRC Translational Immune Discovery Unit, Weatherall Institute of Molecular Medicine, University of Oxford

[andrew.achaiah@ndm.ox.ac.uk](mailto:andrew.achaiah@ndm.ox.ac.uk)

# **Supplementary materials**

## **Methods**

### **Study design**

We performed a retrospective analysis of a cohort of patients (National Health Service Research Ethics approval 14/SC/1060) with an MDT diagnosis of IPF (2018 IPF diagnostic guidelines).^1^ All cases were diagnosed by MDT based on radiological appearance of ILD and clinical history. In this cohort no patients required surgical biopsy for diagnosis. 71 patients from the Oxford Interstitial Lung Disease (ILD) Service with high resolution thoracic CT (HRCT) scans at presentation and at least one further, within the follow-up period of September 2016 - November 2021 were included.

Demographic and comorbidity profiles, time between HRCT scans, pulmonary function tests (PFTs) [Forced Vital Capacity (FVC) and transfer Factor of Lung for Carbon Monoxide (TLco)] closest to CT scans (all within 4 months), and duration of antifibrotic treatment were recorded. Neutrophil, lymphocyte, and monocyte counts were taken from clinical 'full blood count' analysis within 4 months of initial CT.^2^ Annualised change in PFTs and CT scan features, and all-cause mortality rates were collected.

### **CT visual assessment**

Visual assessment of CT has been previously described.^3^ Briefly, a thoracic radiologist with a sub-specialty interest in ILD reviewed each patient’s cross-sectional imaging at MDT to provide a radiological diagnosis that correlated with the patients’ clinical history. All CT abnormalities were defined using standard Fleischner-based terminology and according to 2018 IPF guidelines.^1,4^

Follow-on CTs were classified as either ‘non-progressive’ or ‘progressive’ based upon comparison to first CT. Cases were defined as ‘progressive’ if follow-on CT demonstrated either (i) visual (qualitative) increase in volume of disease (i.e. existing parenchymal features) or (ii) progression of UIP pattern. ‘Non-progressive’ cases were defined as unchanged parenchymal features in follow on CT scan either in terms of (i) extent of these features or (ii) unchanged UIP pattern.

CTs were reported by 1 of 8 thoracic radiologists. All were consultants (with UK Certificate of Completion of Training/CCT for Radiology) and 7 of 8 of these consultants also held a post -CCT Thoracic Radiology Fellowship and participated in dedicated Interstitial Lung Disease MDTs. The other thoracic radiologist has >20 years of experience in reporting HRCT for ILD in our centre. Our thoracic radiologists have contributed to >20 radiology-based studies over the last decade. In one study, performed with our research group, the agreement between of reporting ILD features between the two radiologists was excellent (r=0.91; p<0.001 by Pearson’s Correlation, also tested by Bland Altman; Suppl Fig 4A).^5^

### **CT CALIPER evaluation**

#### **Data processing**

The CALIPER lung texture algorithm (v2.1) was acquired from IMBIO, Minneapolis, USA (www.imbio.com) and installed onto syngo.via software (www.siemens-healthineers.com), a multi-modality reading tool integrated into the local hospital trust system (“client-server platform”). Non-contrast, volumetric, high resolution CT scans (0.625mm slice thickness at an interval of 0.625mm) for appropriate subjects were selected and the Digital imaging and communications in medicine (DICOM) formatted images were uploaded into syngo.via from the local a picture archiving and communications system (PACS).

Initial data processing steps included (i) lung segmentation from adjacent thoracic and chest wall structures, (ii) separation of right and left lungs and (iii) airway segmentation. Lung segmentation is performed using adaptive density-based morphological approach (described in appendix), and airway segmentation involved density thresholding. Right and left lungs were then segmented into upper, middle and lower zones (Figure 3a). The carina was used as a landmark to identify the lower boundary of the upper zone. The remaining two thirds of each lung were equally subdivided into the middle and lower zones for each lung. Total right and left lung volumes were calculated.

#### **Pattern evaluation**

After extracting the pulmonary vessels, parenchymal tissue type classification was applied to 15x15x15 voxel volumes of interest using texture analysis and computer-based algorithmic interpretation of volumetric histogram signature mapping features as previously described above (Figure 3c).^6^ CALIPER evaluation of CT data included classification and quantification of each volume of interest into one of six radiological parenchymal categories: normal lung, hyperlucent, ground glass opacity (GGO), Reticular, honeycombing. Volumes for each parenchymal feature were expressed as a relative percentage of CALIPER-derived (i) total lung volume or (ii) zonal lung volume. Total fibrosis extent represented the sum of GGO, reticular and honeycomb percentages.

The platform provided data output of DICOM series with a colour-coded overlay reflective of parenchymal abnormality and overall disease extent. This was used as a quality control check for each scan. A pdf file was also provided for derived metrics which were then collated into a database for analysis.


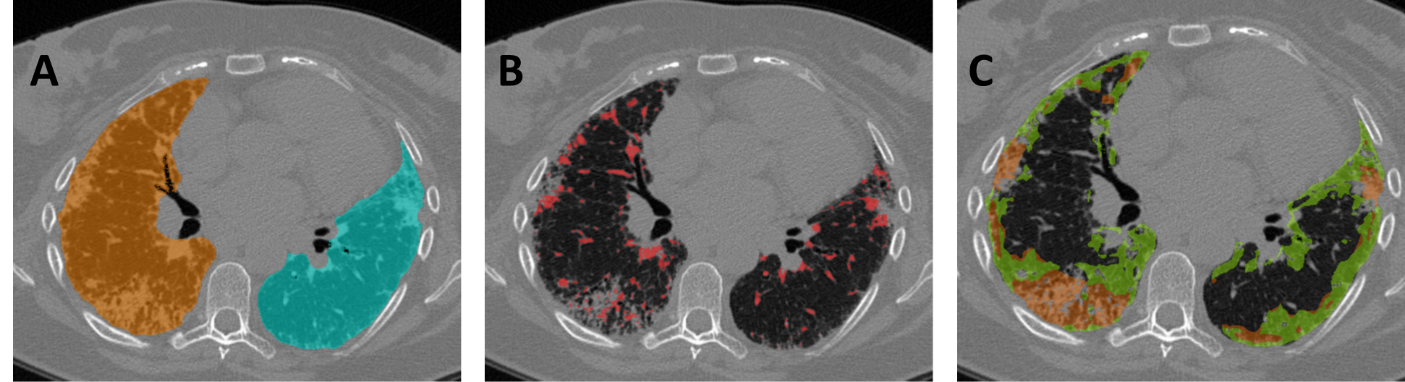


**Figure S1.** Axial slices of CALIPER LTA (A) lung labels, (B) pulmonary vessel labels, (C) lung texture analysis map.

### **Lung function tests**

Spirometry (FEV1 and FVC), Transfer Factor of the Lung for Carbon Monoxide (TLco) and CPI were captured closest to CT. Values are expressed as absolute (FVC; litres, TLco; mmol/min/kPa) or as percentage of predicted value. Lung function trends were calculated as relative (%) change from baseline value. Each follow-up FVC and TLco value (absolute) was divided by the baseline value and multiplied by 100. To draw meaningful comparison to CT trends, annualised trend in lung function trends were deduced from lung function tests recorded closest to first and second CT scan. To calculate annualised trend relative (%) change in FVC and TLco was divided by time (years) between Lung function tests. A measured reduction in absolute forced vital capacity (litres) >10% per year was considered clinically significant and representative of physiological decline.^7^

### **Measurement of disease progression**

Disease progression was measured by absolute change between baseline and follow-up FVC, TLF and CTvol. Annualised percentage change from baseline to follow-up investigation was calculated as:

$$Annualised percentage change (\Delta)=\frac{[(Absolute difference \div Variable at baseline) \times100]}{Time interval between baseline and follow up investigation (Years)}$$

Annualised change was expressed as continuous or categorical variables (>5% or >10%). Composite metrics were made up of different combinations of categorised ∆FVC, ∆TLF and ∆CTvol.

### **Blood leukocyte measurement**

Neutrophil, lymphocyte, and monocyte levels captured using standard 'full blood count' analysis within 4 months of initial CT. monocyte:lymphocyte ratio (MLR), neutrophil:lymphocyte ratio (NLR) and systemic inflammatory response (SIRI) Indexes were calculated as previously described.^3^ Leukocyte values were collated as either continuous or discrete variables. For discrete data, cases were dichotomised by median value leukocyte or index value, or by high or low absolute blood leukocyte (monocyte; < or ≥0.9x10^3^/μl, neutrophils; < or ≥7.5x10^3^/μl, lymphocytes; < or ≥1.0x10^3^/μl) according to local laboratory reference range.

### **Statistical analysis**

Data is expressed as absolute values, relative percentages, means (with standard deviation), medians (interquartile range) or by dichotomised value where stated. Tests for normality of data were performed using a D'Agostino & Pearson test and following this the difference between groups was analysed using paired or unpaired t-tests or Mann-Whitney test for respective parametric and non-parametric analysis. Contingency tests (Fisher’s exact test of significance) were used to assess categorical data.

∆FVC, ∆TLF and ∆CTvol and composite variables were compared against mortality using Cox regression. The censoring time for mortality assessment was the end of follow-up period; 1st January 2022. Multivariate models were adjusted for age, gender, baseline TLF%, antifibrotic duration and leukocytes. Concordance indexes (C-statistic) were used to determine the most discriminate models for predicting mortality.

Reported statistical confidence intervals (CI) are at 95%. Two-tailed p values <0.05 determined statistical significance. All analyses were performed using Graphpad Prism (version 9.2) or SPSS version 27 (IBM Armonk, NY, USA). UpSet plots were used to visualise the relationship and proportions of categorical measurements of disease progression in this cohort using UpSetR Shiny App (<https://gehlenborglab.shinyapps.io/upsetr/>).

# Figures and Tables

## **Figure S2**

**Figure S2.** Annualised disease progression split for all cases achieving composite metric of disease progression (blue) and cases that died during follow up (red).

## **Table S1**

| **Effect of antifibrotic therapy on outcome** | **HR (95% CI)** | **P value** |
| --- | --- | --- |
| **Model: Annualised decline: %Δ FVC reduction >5% or %Δ CALvol >5% or %ΔTLF >5%** | | |
| Age at CT | 1.01 (0.96-1.06) | 0.835 |
| Male | 863701.44 (0-) | 0.983 |
| Total lung fibrosis (%) | 1.03 (1.01-1.06) | 0.015 |
| Annualised decline: %Δ FVC reduction >5% or %Δ CALvol >5% or %ΔTLF >5% | 4.24 (1.02-18.39) | 0.048 |
| Antifibrotic duration (months) | 1.00 (0.99-1.01) | 0.694 |
|  |  |  |
| **Model: Annualised decline: %Δ FVC reduction >5% or %Δ CALvol >5% or %ΔTLF >5%** | | |
| Age at CT | 1.03 (0.98-1.09) | 0.226 |
| Male | 532376.07 (0-) | 0.982 |
| Total lung fibrosis (%) | 1.05 (1.02-1.08) | 0.001 |
| Annualised decline: %Δ FVC reduction >5% or %Δ CALvol >5% or %ΔTLF >5% | 7.14 (2.45-20.79) | <0.001 |
| Antifibrotic duration (months) | 1.00 (0.99-1.01) | 0.994 |
|  |  |  |
| **Model: ΔTLF >10%** | | |
| Age at CT | 1.02 (0.97-1.07) | 0.438 |
| Male | 814037.44 (0-) | 0.981 |
| Total lung fibrosis (%) | 1.04 (1.01-1.06) | 0.010 |
| ΔTLF >10% per year | 6.15 (2.31-16.39) | <0.001 |
| Antifibrotic duration (months) | 1.00 (0.99-1.01) | 0.892 |
|  |  |  |
| **Model: ΔCTvol >10%** | | |
| Age at CT | 1.02 (0.97-1.08) | 0.394 |
| Male | 557632.89 (0-) | 0.983 |
| Total lung fibrosis (%) | 1.04 (1.01-1.07) | 0.004 |
| ΔCTvol >10% | 2.95 (1.26-6.93) | 0.013 |
| Antifibrotic duration (months) | 1.00 (0.99-1.01) | 0.898 |
|  |  |  |
| **Model: ΔFVC >10%** | | |
| Age at CT | 1.02 (0.97-1.08) | 0.394 |
| Male | 557632.89 (0-) | 0.983 |
| Total lung fibrosis (%) | 1.04 (1.01-1.07) | 0.004 |
| ΔFVC >10% | 2.95 (1.26-6.93) | 0.013 |
| Antifibrotic duration (months) | 1.00 (0.99-1.01) | 0.898 |

**Table S1.** Multivariate analysis demonstrating effect of antifibrotic therapy in multivariate modelling.

## **Table S2**

| **Univariate analysis for outcome of mortality** | **HR (95% CI)** | **P value** |
| --- | --- | --- |
| **Demographics** |  |  |
| Age at CT | 1.01 (0.95-1.05) | 0.980 |
| Demographics (Male) | 23.19 (0.04-12852.64) | 0.329 |
| Antifibrotic therapy use | 1.40 (0.62-3.15) | 0.419 |
| Antifibrotic therapy duration (months) | 1.00 (0.99-1.01) | 0.898 |
|  |  |  |
| **Blood leukocytes** |  |  |
| Monocyte | 2.47 (0.37-16.79) | 0.354 |
| Neutrophil | 1.05 (0.91-1.24) | 0.516 |
| Lymphocyte | 0.69 (0.37-1.28) | 0.240 |
|  |  |  |
| **CALIPER (baseline metrics)** |  |  |
| Total lung fibrosis (%) | 1.03 (1.01-1.06) | 0.007 |
| Upper Zone fibrosis % | 1.05 (1.02-1.08) | <0.001 |
| Middle Zone Fibrosis % | 1.02 (1-1.04) | 0.109 |
| Lower Zone fibrosis % | 1.01 (0.99-1.03) | 0.232 |
| TOTAL Ground Glass pct | 1.03 (1.01-1.06) | 0.045 |
| TOTAL Reticular pct | 1.11 (1.03-1.19) | 0.006 |
| TOTAL Honeycombing pct | 1.13 (0.77-1.66) | 0.527 |
| TOTAL Hyperlucent pct | 1.05 (0.98-1.13) | 0.168 |
|  |  |  |
| **Lung function tests (Baseline)** |  |  |
| %FVC | 0.96 (0.93-0.99) | 0.005 |
| %TLCO | 0.95 (0.91-0.98) | 0.004 |
|  |  |  |
| **Disease progression** |  |  |
| Visual evidence of progression on CT | 1.34 (0.48-3.73) | 0.571 |
| ΔCTvol % | 1.83 (1.06-3.17) | 0.031 |
| ΔCTvol >5% | 3.16 (1.25-7.97) | 0.015 |
| ΔCTvol >10% | 2.86 (1.28-6.39) | 0.010 |
| ΔTLF % | 1.04 (1.01-1.09) | 0.035 |
| ΔTLF >5% | 2.03 (1.13-4.53) | 0.042 |
| ΔTLF >10% | 2.41 (1.35-5.90) | 0.041 |
| ΔFVC % | 0.98 (0.96-0.99) | 0.009 |
| ΔFVC >5% | 3.19 (1.3-7.84) | 0.012 |
| ΔFVC >10% | 5.16 (2.18-12.22) | <0.001 |
| ΔFVC>5% or ΔCTvol>5% or ΔTLF% >5% | 3.16 (1.21-10.61) | 0.032 |
| ΔFVC>10% or ΔCTvol>10% or ΔTLF%>10% | 5.08 (2.01-12.89) | <0.001 |
| ΔFVC>5% & ΔCTvol>5% & ΔTLF>5% | 3.08 (1.32-7.21) | 0.010 |
| ΔFVC>10% & ΔCTvol>10% & ΔTLF>10% | 5.62 (1.61-19.8) | 0.007 |
| ΔFVC>5% & ΔTLF>5% | 2.81 (1.23-6.43) | 0.015 |
| ΔFVC>10% & ΔTLF >10% | 3.4 (1.15-10.08) | 0.027 |

**Table S2**. Univariate Cox regression for mortality. P<0.05 considered statistically significant.

# **Declarations**

**Ethical Approval**

Ethical Approval was granted by National Health Service Research Ethics approval 14/SC/1060.

**Consent for publication**

No identifying images or other personal or clinical details of participants are presented that compromise anonymity. Patient consent to publication is Not Applicable with respect to this manuscript.

**Funding**

The study was funded by the National Institute for Health Research (NIHR) Oxford Biomedical Research Centre (BRC). LPH is supported in part by MRC UK (MC_UU_00008/1). AA was funded by Oxford BRC.

**Availability of data and materials**

All data generated or analysed during this study are included in this published article and supplementary information files.

**Acknowledgement**

The study was funded by the National Institute for Health Research (NIHR) Oxford Biomedical Research Centre (BRC). LPH is supported in part by MRC UK (MC_UU_00008/1). AA was funded by Oxford BRC.

**Authors contribution**

AA conceived project, conducted analysis, interpreted data, and wrote the paper. EF, PS, RB and RH provided guidance with project. LPH conceived project, interpreted data, wrote paper, and supervised the study.

**Conflict of interest disclosures:**

Andrew Achaiah, Emily Fraser, Rachel Benamore, Ling-Pei Ho and Rachel Hoyles report no relevant conflict of interests. Peter Saunders has received consultancy fees from Trevi Therapeutics, and lecture fees from Boehringer Ingelheim, but no other conflict of interest.

**Patient and Public Involvement:**

Patient and public were not involved in design, recruitment, conduct of this study.

# **References**

1. Raghu G, Remy-Jardin M, Myers JL, et al. Diagnosis of Idiopathic Pulmonary Fibrosis. An Official ATS/ERS/JRS/ALAT Clinical Practice Guideline. *Am J Respir Crit Care Med*. Sep 1 2018;198(5):e44-e68. doi:10.1164/rccm.201807-1255ST

2. Scott MKD, Quinn K, Li Q, et al. Increased monocyte count as a cellular biomarker for poor outcomes in fibrotic diseases: a retrospective, multicentre cohort study. *Lancet Respir Med*. Jun 2019;7(6):497-508. doi:10.1016/s2213-2600(18)30508-3

3. Achaiah A, Rathnapala A, Pereira A, et al. Neutrophil lymphocyte ratio as an indicator for disease progression in Idiopathic Pulmonary Fibrosis. *BMJ Open Respiratory Research*. 2022;9(1):e001202. doi:10.1136/bmjresp-2022-001202

4. Hansell DM, Bankier AA, MacMahon H, McLoud TC, Müller NL, Remy J. Fleischner Society: glossary of terms for thoracic imaging. *Radiology*. Mar 2008;246(3):697-722. doi:10.1148/radiol.2462070712

5. Benamore R, Kendrick YR, Repapi E, et al. CTAS: a CT score to quantify disease activity in pulmonary sarcoidosis. *Thorax*. 2016;71(12):1161-1163. doi:10.1136/thoraxjnl-2016-208833

6. Bartholmai BJ, Raghunath S, Karwoski RA, et al. Quantitative computed tomography imaging of interstitial lung diseases. *J Thorac Imaging*. Sep 2013;28(5):298-307. doi:10.1097/RTI.0b013e3182a21969

7. Richeldi L, Ryerson CJ, Lee JS, et al. Relative versus absolute change in forced vital capacity in idiopathic pulmonary fibrosis. *Thorax*. May 2012;67(5):407-11. doi:10.1136/thoraxjnl-2011-201184
